# Insight into the characteristics of research published in traditional, complementary, alternative, and integrative medicine journals: a bibliometric analysis

**DOI:** 10.1186/s12906-021-03354-7

**Published:** 2021-07-01

**Authors:** Jeremy Y. Ng

**Affiliations:** 1 Centre for Journalology, Ottawa Methods Centre, Ottawa Hospital Research Institute, The Ottawa Hospital, General Campus, Centre for Practice Changing Research Building, 501 Smyth Road, PO BOX 201B, Ottawa, ON K1H 8L6 Canada; 2 Department of Health Research Methods, Evidence, and Impact, Faculty of Health Sciences, McMaster University, Michael G. DeGroote Centre for Learning and Discovery, Room 2112, 1280 Main Street West, Hamilton, ON L8S 4K1 Canada

**Keywords:** Bibliometric analysis, Complementary and alternative medicine, Integrative health, Integrative medicine, Research trends, Scientometrics

## Abstract

**Background:**

Traditional, complementary, alternative and integrative medicine (TCAIM) can be described as diverse medical and healthcare interventions, practices, products, or disciplines that are not considered as part of conventional medicine. Inherent in its definition, TCAIMs are comprised of a wide variety of therapies with highly variable safety and effectiveness evidence profiles. Despite this, the use of many TCAIMs is highly prevalent among patients globally. The present study consists of a bibliometric analysis of TCAIM journals.

**Methods:**

A single search of all International Standard Serial Number (ISSNs) of all journals categorized as “complementary and alternative medicine” (code 2707) based on the All Science Journal Classification (ASJC) was run on Scopus on April 17, 2021. All publication types were included; no further search limits were applied. The following bibliometric data were collected: number of publications (in total and per year), authors and journals; open access status; journals publishing the highest volume of literature and their impact factors; language, countries, institutional affiliations, and funding sponsors of publications; most productive authors; and highest-cited publications. Trends associated with this subset of publications were identified and presented. Bibliometric indicators of production were calculated, and bibliometric networks were constructed and visualized using the software tool VOSviewer.

**Results:**

A total of 172,466 publications (42,331 open access), were published by 219,680 authors in 143 journals from 1938 to 2021. Since the 1940s, an upward trend with respect to the volume of publications can be observed, with a steep increase observed between the mid-2000s and mid-2010s. The journal that published the largest number of publications was the Journal of Natural Products (*n* = 15,144). The most productive countries included China (*n* = 45,860), the United States (*n* = 29,523), and Germany (*n* = 10,120); a number of the most common institutional affiliations and funding sponsors also originated from these three countries.

**Conclusions:**

The number of publications collectively published in TCAIM journals follows an upward trend. Given a high prevalence of TCAIM use among patients, increased acceptance of TCAIM among conventional healthcare providers, and growing interest in the research of TCAIM, future work should continue to investigate and track changes in the publication characteristics of the emerging research on this topic.

## Background

Complementary and alternative medicine is generally defined as a group of diverse medical and healthcare interventions, practices, products or disciplines that are not considered as part of conventional medicine [1]. Specifically, the National Center for Complementary and Integrative Health (NCCIH) defines “complementary” medicine as a non-mainstream practice used *together with* conventional medicine, whereas “alternative” medicine refers to a non-mainstream practice used *in place of* conventional medicine [2]. In contrast, “integrative health” is defined as the coordinated delivery of conventional and complementary approaches together [2]. These three words – complementary, alternative, and integrative – comprise the most common ways to refer to these types of therapies [3], in additional to “traditional medicine” which has been defined by the World Health Organization as “the sum total of the knowledge, skill and practices based on the theories, beliefs and experiences indigenous to different cultures, whether explicable or not, used in the maintenance of health as well as in the prevention, diagnosis, improvement or treatment of physical and mental illness” [4]. For the purpose of the present study, these therapies will be referred to collectively as “traditional, complementary, alternative and integrative medicine” or “TCAIMs” hereafter. At present, a lack of consensus exists regarding how to categorize TCAIMs; in fact, by definition every therapy that falls under the umbrella of “TCAIM” exists as a result of being considered outside of the purview of conventional Western medical practices [3]. Therefore, one TCAIM therapy can be highly unrelated to another due to the fact that each originates from a different region in the world, culture, system of traditional medicine, and school of thought [4, 5]. Despite these challenges, attempts have been made to categorize TCAIMs. For example, the NCCIH divides TCAIM therapies into two main types: 1) natural products and 2) mind and body practices. Within the former category, they include therapies such as herbs, vitamins and minerals, and probiotics, while in the latter, they include therapies such as yoga, chiropractic and osteopathic manipulation, and meditation, as well as acupuncture, relaxation techniques, tai chi, qi gong, and hypnotherapy, among others [2]. Despite these efforts, the NCCIH has stated, however, that some TCAIMs may not fit neatly into either of these two groups, citing many systems of TCAIM including practices of traditional healers, Ayurvedic medicine, traditional Chinese medicine, homeopathy, naturopathy, and functional medicine [2].

Regardless of how TCAIMs are categorized, these therapies are perceived to be of value by their proponents for their emphasis on a holistic, patient-focused approach to healthcare, which include mental, emotional, functional, spiritual, economic, and social aspects [2, 6]. TCAIM is widely used around the world, with 88% of World Health Organization member states acknowledging their use, which by definition means that these 170 countries have formally developed policies, laws, regulations, programs and offices for TCAIM [4]. The prevalence of TCAIM use is high in many Western countries; for example, it is estimated that around 80% of Canadians have used TCAIM [7]. The prevalence of TCAIM use is also documented to be high among certain patient populations; in cancer patients, as many as 90% report using some type of TCAIM [8–10]. TCAIM is used by these patients for a variety of reasons, including symptom relief, improved quality of life, supplementing conventional therapy, supporting one’s philosophical orientations toward health, and achieving a sense of control over one’s care [11–13]. Integrative medicine (the use of complementary and conventional therapies) is becoming increasingly popular among patients and practitioners [14, 15], and sub-specializations of integrative care for specific diseases/conditions have also been established, such as integrative oncology [16, 17].

While some TCAIMs, such as meditation and yoga [18, 19], have undergone more thorough testing and have been found to be generally safe and effective, others have not been adequately researched to determine their effectiveness, and some have been found to be potentially harmful or interact negatively with conventional medicines [20–22]. The belief among patients that “natural means safe and better” [23] is well-documented, however, evidence from the research literature suggests otherwise. Many herbal and dietary supplements can be harmful when taken in large quantities. Certain weight loss and bodybuilding supplements have been shown to cause hepatotoxicity or even hepatic failure at therapeutic doses [24]. Furthermore, systemized pharmacovigilance of TCAIMs is poorly coordinated on a national and international level, and TCAIM therapies are generally not held to the same standards of regulation as that of pharmaceutical medicines in terms of quality, effectiveness, and safety [20].

The increase in popularity and prevalence of TCAIM use among patients, and growing acknowledgement among conventional healthcare providers that a need exists to approach TCAIM therapies, their traditions, and their practitioners with respect, are among some of the reasons for an increase in TCAIM research productivity which has resulted in a growth in the volume of the published literature over the past few decades [25–27]. The application of a research method known as a bibliometric analysis can facilitate a better understanding of a given field, such as that of TCAIM. A bibliometric analysis involves the statistical assessment of scientific publications, to identify the characteristics and determine the impact of the literature published in a specific academic discipline [28–30]. This increased interest in TCAIM research has led to the establishment and indexing of multiple TCAIM journals. While a number of bibliometric analyses have made attempts to evaluate the characteristics of all publications published in the area of traditional, complementary, alternative, and/or integrative medicine through the use of various search strategies [31–35], no study has comprehensively assessed the characteristics of the publications found within these source titles to date. Thus, the purpose of the present study is to provide current insight into the characteristics of publications published across TCAIM journals through a bibliometric analysis.

## Methods

### Publication search and characteristics

The 2021 Scopus Source List [36] was downloaded, and all Scopus-indexed journals belonging to the “complementary and alternative medicine” category (code 2707) were identified based on the All Science Journal Classification (ASJC). A single search containing the International Standard Serial Numbers (ISSNs) of all of these journals was run on Scopus on April 17, 2021; the search strategy can be found in Table 1. Search results were exported on the same day to prevent discrepancies between daily database updates. Searches were only conducted on Scopus because it is the largest abstract and citation database of peer-reviewed literature [37]; in comparison, Web of Science contains considerably fewer TCAIM-categorized journals, while OVID databases do not provide certain metrics such as publication citation counts [38]. All publication types were included, and no further search limits were applied. The following bibliometric data were collected: number of publications (in total and per year), authors and journals; open access status; journals publishing the highest volume of literature and their impact factors; language, countries, institutional affiliations, and funding sponsors of publications; most productive authors; and highest-cited publications. Trends associated with this subset of publications were identified and presented. Bibliometric networks were constructed and visualized using the software tool VOSviewer (version 1.6.16) [39, 40]. All aforementioned steps were conducted by a single author (JYN).

**Table 1:** Scopus Search Strategy Executed on April 17, 2021

ISSN (23755776) OR ISSN (03601293) OR ISSN (09645284) OR ISSN (22129588) OR ISSN (26624052) OR ISSN (01896016) OR ISSN (16146891) OR ISSN (10762809) OR ISSN (10814000) OR ISSN (1096942X) OR ISSN (10895159) OR ISSN (10786791) OR ISSN (15223396) OR ISSN (00913960) OR ISSN (0192415X) OR ISSN (00029157) OR ISSN (17535174) OR ISSN (16148339) OR ISSN (1573420X) OR ISSN (18339735) OR ISSN (2209119X) OR ISSN (10338330) OR ISSN (10338330) OR ISSN (14726882) OR ISSN (07177917) OR ISSN (00070785) OR ISSN (16720415) OR ISSN (20956975) OR ISSN (17498546) OR ISSN (02532670) OR ISSN (09302786) OR ISSN (2045709X) OR ISSN (17461340) OR ISSN (10360913) OR ISSN (08896976) OR ISSN (14611449) OR ISSN (15332101) OR ISSN (02684055) OR ISSN (25042092) OR ISSN (17443881) OR ISSN (09652299) OR ISSN (13536117) OR ISSN (22150838) OR ISSN (04156412) OR ISSN (18763820) OR ISSN (13516647) OR ISSN (1741427X) OR ISSN (11762330) OR ISSN (15508307) OR ISSN (14653753) OR ISSN (16614119) OR ISSN (00180599) OR ISSN (08879311) OR ISSN (14754916) OR ISSN (09747168) OR ISSN (09725938) OR ISSN (15347354) OR ISSN (1546993X) OR ISSN (23252812) OR ISSN (11773936) OR ISSN (22134220) OR ISSN (19406223) OR ISSN (09624562) OR ISSN (10471979) OR ISSN (19826206) OR ISSN (17460689) OR ISSN (19723539) OR ISSN (09750185) OR ISSN (20052901) OR ISSN (16723597) OR ISSN (10755535) OR ISSN (10286020) OR ISSN (09759476) OR ISSN (22311866) OR ISSN (13608592) OR ISSN (15446301) OR ISSN (01438042) OR ISSN (15563499) OR ISSN (15533840) OR ISSN (14468263) OR ISSN (17464269) OR ISSN (21565872) OR ISSN (13094572) OR ISSN (12268453) OR ISSN (22108033) OR ISSN (10496475) OR ISSN (19960875) OR ISSN (16840240) OR ISSN (18610293) OR ISSN (01633864) OR ISSN (08344825) OR ISSN (20936966) OR ISSN (10841288) OR ISSN (13263390) OR ISSN (1715894X) OR ISSN (22254110) OR ISSN (18801447) OR ISSN (18638678) OR ISSN (00252514) OR ISSN (19336586) OR ISSN (11239395) OR ISSN (15763080) OR ISSN (07346875) OR ISSN (1934578X) OR ISSN (22103155) OR ISSN (19408153) OR ISSN (08098131) OR ISSN (08039828) OR ISSN (09747877) OR ISSN (15982386) OR ISSN (16159071) OR ISSN (13880209) OR ISSN (09737847) OR ISSN (09580344) OR ISSN (09723293) OR ISSN (09447113) OR ISSN (16248597) OR ISSN (16286847) OR ISSN (00320943) OR ISSN (18193455) OR ISSN (15160572) OR ISSN (15760952) OR ISSN (18878369) OR ISSN (18888526) OR ISSN (18789730) OR ISSN (10150684) OR ISSN (10950656) OR ISSN (15564061) OR ISSN (13021192) OR ISSN (23264500) OR ISSN (09735070) OR ISSN (00986151) OR ISSN (1560604X) OR ISSN (26160684) OR ISSN (23118571) OR ISSN (0722348X) OR ISSN (22129596) OR ISSN (26624060) OR ISSN (15734218) OR ISSN (22091203) OR ISSN (26627671) OR ISSN (23788763) OR ISSN (19930402) OR ISSN (18755364) OR ISSN (25042106) OR ISSN (22150846) OR ISSN (14394359) OR ISSN (17414288) OR ISSN (16614127) OR ISSN (15505138) OR ISSN (14764245) OR ISSN (23207094) OR ISSN (9751068) OR ISSN (22134239) OR ISSN (15322106) OR ISSN (19930399) OR ISSN (14772213) OR ISSN (9762809) OR ISSN (22311874) OR ISSN (2515690X) OR ISSN (21463298) OR ISSN (20934947) OR ISSN (22108041) OR ISSN (15403580) OR ISSN (21468397) OR ISSN (15206025) OR ISSN (22346856) OR ISSN (20957548) OR ISSN (18813747) OR ISSN (14330466) OR ISSN (15559475) OR ISSN (22103163) OR ISSN (22111069) OR ISSN (17445116) OR ISSN (9762787) OR ISSN (10991565) OR ISSN (17652847) OR ISSN (14390221) OR ISSN (19885806) OR ISSN (23264519) OR ISSN (26160692) OR ISSN (10035257) OR ISSN (25892894) OR ISSN (16721977) OR ISSN (10015302)	

The journal titles associated with each ISSN provided in this search strategy is provided in Table 2

### Bibliometric indicators of production

Relative growth rates and doubling times were calculated for publications published between 1938 and 2020. The relative growth rate represents the increase in the number of publications published per unit of time. The relative growth rate was calculated based on the following equation: [*Relative Growth Rate = (log*_*e*_*W*_*2*_ *- log*_*e*_*W*_*1*_*)/(T2 - T1)],* where log_e_ W_1_ represents the log of initial number of articles, and log_e_ W_2_ represents the log of final number of articles after a specific period of interval. T2-T1 represents the unit difference between the initial time and the final time. Doubling time is defined as the amount of time required for the subject matter to double its production. The doubling time was calculated based on the following equation: [*DT = 0.693/Relative Growth Rate]*. Price’s law was also applied to the subset of publications analysed [41]. This law proposes that the growth of scientific production follows an exponential function, and represents one of the most common indicators used to analyse productivity in a specific discipline or subset of publications. To assess whether the increase in data conforms to Price’s law of exponential growth, we carried out a linear adjustment of the values and another adjustment to an exponential curve.

## Results

A total of 172,466 publications (42,331 open access), were published by 219,680 unique authors in 143 journals from 1938 to 2021. Since the 1940s, an upward trend with respect to the volume of publications can be observed, with a steep increase observed between the mid-2000s and mid-2010s. This upward trend has continued with 2020 marking the year with the highest number of publications to date. The Journal of Natural Products (*n* = 15,144) published the largest number of publications indexed in Scopus, followed by Zhongguo Zhongyao Zazhi (*n* = 14,577), and Planta Medica (*n* = 10,793). All journals included within this bibliometric analysis were hand-searched on InCites Journal Citation Reports [42]. As of 2020, 83 journals were still active (57.6%), of which 35 had a 2019 impact factor (range from 0.200 to 5.487). Table 2 provides complete details of the journals included in this bibliometric analysis, including the journal name, ISSN, whether the journal is active or inactive (as of 2020), coverage period, title history indication, publisher name, number of publications indexed in Scopus, and the 2019 impact factor (if available).

**Table 2:** Characteristics of TCAIM Journals Indexed in Scopus (*n* = 143)

Position	Journal Name	ISSN	Active or Inactive (as of 2020)	Coverage Period	Title History Indication	Publisher Name	Number of Publications Indexed in Scopus	2019 Impact Factor
1	Journal of Natural Products	1633864 (Print); 15206025 (Electronic)	Active	1978-ongoing, 1973, 1971, 1949	Formerly known as Lloydia; not categorized as “complementary and alternative medicine” by Scopus	American Chemical Society	15144	3.782
2	Zhongguo Zhongyao Zazhi	10015302 (Print)	Active	1989-ongoing	Formerly known as Zhong Yao Tong Bao (Beijing, China: 1981); not categorized as “complementary and alternative medicine” by Scopus	Zhongguo Zhongyi Yanjiuyuan	14577	N/A
3	Planta Medica	00320943 (Print); 14390221 (Electronic)	Active	1965-ongoing, 1961	N/A	Georg Thieme Verlag	10793	2.687
4	The Journal of the American Osteopathic Association	986151 (Print)	Active	1945-ongoing	N/A	American Osteopathic Association	9533	N/A
5	Evidence-Based Complementary and Alternative Medicine	1741427X (Print); 17414288 (Electronic)	Active	2005-ongoing	N/A	Hindawi Publishing Corporation	9261	1.813
6	Chinese Traditional and Herbal Drugs	02532670 (Print)	Active	2006-ongoing	N/A	Chung Tsao Yao Tsa Chih Pien Chi Pu	8704	N/A
7	Natural Product Communications	1934578X (Print); 15559475 (Electronic)	Active	2008-ongoing	N/A	SAGE Publications Inc.	5800	0.468
8	Pharmaceutical Biology	13880209 (Print); 17445116 (Electronic)	Active	1975-ongoing, 1961-1972	Formerly known as International Journal of Pharmacognosy; not categorized as “complementary and alternative medicine” by Scopus	Taylor & Francis	4901	2.971
9	Phytomedicine	09447113 (Print)	Active	1994-ongoing	N/A	Elsevier BV	4212	4.268
10	BMC Complementary and Alternative Medicine	14726882 (Print)	Inactive	2001-2019	Continued as BMC Complementary Medicine and Therapies; see position #63	BioMed Central	3902	N/A (Inactive)
11	Journal of Alternative and Complementary Medicine	10755535 (Print)	Active	1995-ongoing	N/A	Mary Ann Liebert Inc.	3709	2.256
12	Journal of Asian Natural Products Research	10286020 (Print); 14772213 (Electronic)	Active	1998-ongoing	N/A	Taylor & Francis	2822	1.345
13	American Journal of Chinese Medicine	0192415X (Print)	Active	1979-ongoing, 1974-1977	Formerly known as Comparative Medicine East and West; not categorized as “complementary and alternative medicine” by Scopus	World Scientific Publishing Co	2719	3.682
14	British Homeopathic Journal	00070785 (Print)	Inactive	1998-2001, 1945-1995	Continued as Homeopathy: The Journal of the Faculty of Homeopathy; see position #44	Elsevier BV	2652	N/A (Inactive)
15	American Journal of Clinical Hypnosis	00029157 (Print)	Active	1958-ongoing	N/A	Taylor and Francis Inc.	2573	0.766
16	Complementary Therapies in Medicine	09652299 (Print)	Active	1993-ongoing	Formerly known as Complementary Medical Research; see position #123	Churchill Livingstone	2436	2.063
17	Chinese Journal of Integrative Medicine	16720415 (Print); 19930402 (Electronic)	Active	2005-ongoing	Formerly known as Chinese Journal of Integrated Traditional and Western Medicine; not categorized as “complementary and alternative medicine” by Scopus	Springer Nature Switzerland AG	2170	1.545
18	Manuelle Medizin	00252514 (Print); 14330466 (Electronic)	Active	1973-ongoing	N/A	Springer Verlag	2003	N/A
19	Journal of Bodywork and Movement Therapies	13608592 (Print)	Active	1996-ongoing	N/A	Churchill Livingstone	1987	N/A
20	Phytochemical Analysis	09580344 (Print); 10991565 (Electronic)	Active	1990-ongoing	N/A	John Wiley & Sons Inc.	1954	2.772
21	Alternative Therapies in Health and Medicine	10786791 (Print)	Active	1995-ongoing	N/A	InnoVision Communications	1892	0.937
22	Zhong Xi Yi Jie He Xue Bao = Journal of Chinese integrative medicine	16721977 (Print)	Inactive	2003-2012	Continued as Journal of Integrative Medicine; not categorized as “complementary and alternative medicine” by Scopus	Shanghai Association of Integrative Medicine	1861	N/A (Inactive)
23	Holistic Nursing Practice	08879311 (Print); 15505138 (Electronic)	Active	1986-ongoing	Formerly known as Topics in Clinical Nursing; not categorized as “complementary and alternative medicine” by Scopus	Lippincott Williams & Wilkins Ltd.	1722	0.968
24	Zeitschrift für Phytotherapie: Offizielles Organ der Ges. f. Phytotherapie e.V	0722348X (Print)	Active	1985-ongoing, 1982	N/A	Hippokrates Verlag	1648	N/A
25	Alternative and Complementary Therapies	10762809 (Print)	Active	1999-ongoing	N/A	Mary Ann Liebert Inc.	1631	N/A
26	Journal of Medicinal Plant Research	19960875 (Print)	Inactive	2009-2011	N/A	Academic Journals	1495	N/A (Inactive)
27	EXPLORE: The Journal of Science and Healing	15508307 (Print)	Active	2005-ongoing	N/A	Elsevier BV	1462	1.485
28	Journal of Natural Medicines	18610293 (Print)	Active	2006-ongoing	Formerly known as Natural Medicines; not categorized as “complementary and alternative medicine” by Scopus	Springer Verlag	1461	2.055
29	Indian Journal of Traditional Knowledge	09725938 (Print); 09751068 (Electronic)	Active	2008-ongoing	N/A	National Institute of Science Communication and Information Resources	1446	0.731
30	Acupuncture in Medicine	09645284 (Print)	Active	1996-ongoing	N/A	BMJ Publishing Group	1391	2.129
31	Deutsche Zeitschrift für Akupunktur	04156412 (Print); 14394359 (Electronic)	Inactive	1984-2016 (cancelled)	N/A	Springer Medizin	1344	N/A (Inactive)
32	Integrative Cancer Therapies	15347354 (Print)	Active	2002-ongoing	N/A	Sage Science Press	1258	2.379
33	Complementary Therapies in Clinical Practice	17443881 (Print)	Active	2005-ongoing	Formerly known as Complementary Therapies in Nursing and Midwifery; see position #76	Elsevier BV	1231	1.770
34	Focus on Alternative and Complementary Therapies	14653753 (Print)	Inactive	2004-2016	N/A	Wiley-Blackwell	1165	N/A (Inactive)
35	Journal of Acupuncture and Tuina Science	16723597 (Print); 19930399 (Electronic)	Active	2007-ongoing	N/A	Springer Nature Switzerland AG	1156	N/A
36	Chinese Journal of Natural Medicines	20956975 (Print); 18755364 (Electronic)	Active	2004-ongoing	N/A	China Pharmaceutical University	1147	2.014
37	European Journal of Integrative Medicine	18763820 (Print)	Active	2008-ongoing	N/A	Elsevier BV	1120	0.974
38	Revista Brasileira de Plantas Medicinais	15160572 (Print)	Inactive	1999-2016	N/A	Fundacao do Instituto de Biociencias	1115	N/A (Inactive)
39	Journal of Medicinal Plants	16840240 (Print)	Active	2004-ongoing	N/A	Pizhuhishkadh-i giyahan-i darayiva faravardah ha-vi tabbii	1090	N/A
40	Phytotherapie	16248597 (Print); 17652847 (Electronic)	Active	2005-ongoing	N/A	Springer Verlag	1071	N/A
41	African Journal of Traditional, Complementary and Alternative Medicines	01896016 (Print)	Inactive	2006-2016 (cancelled), 2002, 1995, 1991, 1981-1982, 1973-1978, 1970	N/A	African Networks on Ethnomedicines	995	N/A (Inactive)
42	Forschende Komplementarmedizin	16614119 (Print); 16614127 (Electronic)	Inactive	2006-2016, 2002	Formerly known as Forschende Komplementarmedizin und Klassische Naturheilkunde; not categorized as “complementary and alternative medicine” by Scopus	S. Karger AG	990	N/A (Inactive)
43	Schweizerische Zeitschrift für GanzheitsMedizin	10150684 (Print)	Inactive	2002-2017	N/A	Dr. Becker & Partner AG-Verlag fuer Ganzheits Medizin	934	N/A (Inactive)
44	Homeopathy: The Journal of the Faculty of Homeopathy	14754916 (Print); 14764245 (Electronic)	Active	1998-ongoing	Formerly known as British Homeopathic Journal; see position #14	Churchill Livingstone	934	N/A
45	Journal of Ethnobiology and Ethnomedicine	17464269 (Print)	Active	2005-ongoing	N/A	BioMed Central	928	2.264
46	Journal of Herbs, Spices and Medicinal Plants	10496475 (Print); 15403580 (Electronic)	Active	1992-ongoing	N/A	The Haworth Herbal Press	922	N/A
47	American Journal of Acupuncture	00913960 (Print)	Inactive	1975-1999	N/A	American Journal of Acupuncture	843	N/A (Inactive)
48	Acupuncture and Electro-Therapeutics Research	03601293 (Print)	Active	1976-ongoing	N/A	Cognizant Communication Corporation	838	0.200
49	Medical Acupuncture	19336586 (Print)	Active	2008-ongoing	N/A	Mary Ann Liebert Inc.	825	N/A
50	Journal of Ginseng Research	12268453 (Print); 20934947 (Electronic)	Active	2010-ongoing	N/A	Elsevier BV	770	5.487
51	Boletin Latinoamericano y del Caribe de Plantas Medicinales y Aromaticas	07177917 (Print)	Active	2008-ongoing	N/A	Universidad de Santiago de Chile	701	0.819
52	Journal of Complementary and Integrative Medicine	15533840 (Print)	Active	2006-ongoing	N/A	Walter de Gruyter GmbH	699	N/A
53	Journal of Ayurveda and Integrative Medicine	09759476 (Print); 09762809 (Electronic)	Active	2010-ongoing	N/A	Elsevier BV	690	N/A
54	Chinese Medicine	17498546 (Print)	Active	2006-ongoing	N/A	BioMed Central	619	2.960
55	Integrative Medicine	1546993X (Print)	Active	2013-ongoing, 2005-2010	Formerly known as International Journal of Integrative Medicine; not categorized as “complementary and alternative medicine” by Scopus	InnoVision Communications	611	N/A
56	Alternative Medicine Review	10895159 (Print)	Inactive	1996-2012	N/A	Thorne Reasearch Inc.	587	N/A (Inactive)
57	Journal of Traditional and Complementary Medicine	22254110 (Print)	Active	2011-ongoing	N/A	Elsevier BV	562	N/A
58	JAMS Journal of Acupuncture and Meridian Studies	20052901 (Print)	Active	2008-ongoing	N/A	Elsevier BV	531	N/A
59	Chiropractic and Manual Therapies	2045709X (Print)	Active	2011-ongoing	Formerly known as Chiropractic and Osteopathy; see position #113	BioMed Central	475	1.512
60	Osteopathische Medizin	16159071 (Print)	Inactive	2004-2017 (cancelled)	N/A	Elsevier BV	470	N/A (Inactive)
61	Medicina Naturista	15763080 (Print)	Active	2012-ongoing	N/A	Zaragoza: Universidad de Zaragoza, Facultad de Medicina	462	N/A
62	Journal of Chinese Medicine	01438042 (Print)	Active	2016-ongoing, 2001-2013	N/A	Eastland Press	459	N/A
63	BMC Complementary Medicine and Therapies	26627671 (Electronic)	Active	2020-ongoing	Formerly known as BMC Complementary and Alternative Medicine; see position #10	BioMed Central Ltd.	456	2.833
64	International Journal of Osteopathic Medicine	17460689 (Print)	Active	2005-ongoing	N/A	Elsevier Ltd	454	1.208
65	Revista Internacional de Acupuntura	18878369 (Print)	Active	2007-ongoing	N/A	Elsevier BV	452	N/A
66	Tropical Journal of Natural Product Research	26160684 (Print); 26160692 (Electronic)	Active	2017-ongoing	N/A	Faculty of Pharmacy, University of Benin	452	N/A
67	Nordic Journal of Music Therapy	08098131 (Print)	Active	2001-ongoing	Formerly known as Nordisk Tidsskrift for Musikkterapi; see position #104	Taylor & Francis	442	0.913
68	Chinesische Medizin	09302786 (Print)	Inactive	1999-2017 (cancelled)	N/A	Springer International Publishing AG	437	N/A (Inactive)
69	International Journal of Phytomedicine	09750185 (Print)	Inactive	2010-2016 (cancelled)	N/A	Advanced Research Journals	427	N/A (Inactive)
70	Revue d'Homeopathie	18789730 (Print)	Active	2010-ongoing	N/A	Elsevier Masson	416	N/A
71	Studies on Ethno-Medicine	09735070 (Print)	Active	2009-ongoing	N/A	Kamla-Raj Enterprises	406	N/A
72	Journal of Herbal Medicine	22108033 (Print); 22108041 (Electronic)	Active	2011-ongoing	N/A	Urban und Fischer Verlag Jena	405	2.221
73	Journal of Biologically Active Products from Nature	22311866 (Print); 22311874 (Electronic)	Active	2011-ongoing	N/A	Taylor and Francis Ltd.	402	N/A
74	Natural Products Journal	22103155 (Print); 22103163 (Electronic)	Active	2011-ongoing	N/A	Bentham Science Publishers B.V.	395	N/A
75	Music Therapy Perspectives	07346875 (Print)	Active	2011-ongoing	N/A	Oxford University Press	391	N/A
76	Complementary Therapies in Nursing and Midwifery	13536117 (Print)	Inactive	1995-2004	Continued as Complementary Therapies in Clinical Practice; see position #33	Elsevier BV	385	N/A (Inactive)
77	Complementary Health Practice Review	15332101 (Print)	Inactive	2007-2010, 1996-1999	Continued as Journal of Evidence-Based Complementary and Alternative Medicine; see positions #79 and #125	SAGE Publications Inc.	375	N/A (Inactive)
78	Integrative Medicine Alert	23252812 (Print)	Active	2013-ongoing	Formerly known as Alternative Medicine Alert; see position #99	American Health Consultants, Inc.	374	N/A
79	Journal of Evidence-Based Complementary and Alternative Medicine	21565872 (Print)	Inactive	2014-2018, 1995	Continued as Journal of Evidence-Based Integrative Medicine; see positions #77, #125 and #136	SAGE Publications	369	N/A (Inactive)
80	Oriental Pharmacy and Experimental Medicine	15982386 (Print); 22111069 (Electronic)	Inactive	2012-2019	Continued as Advances in Traditional Medicine; see position #108	Springer Science + Business Media	357	N/A (Inactive)
81	KIM - Komplementare und Integrative Medizin, Artztezeitschrift für Naturheilverfahren	18638678 (Print)	Inactive	2007-2009	Formerly known as Arztezeitschrift fur Naturheilverfahren und Regulationsmedizin; see position #91	Urban & Fischer Verlag	336	N/A (Inactive)
82	Complementary Medicine Research	25042092 (Print); 25042106 (Electronic)	Active	2017-ongoing, 2015	N/A	S. Karger AG	314	1.089
83	Research Journal of Medicinal Plant	18193455 (Print)	Inactive	2009-2016 (cancelled)	N/A	Academic Journals Inc.	311	N/A (Inactive)
84	International Journal of Aromatherapy	09624562 (Print); 15322106 (Electronic)	Inactive	1995-2006	N/A	Essential Oil Resource Consultants	307	N/A (Inactive)
85	International Journal of High Dilution Research	19826206 (Print)	Active	2011-ongoing	N/A	Universidade Estadual Paulista - UNESP	300	N/A
86	Herba Polonica	00180599 (Print)	Active	2018-ongoing, 1973-1979	N/A	Instytut Roslin i Przetworow Zielarskich	284	N/A
87	Sleep and Hypnosis	13021192 (Print)	Active	2000-ongoing	N/A	Kure Iletisim Grubu A S	282	N/A
88	Thermology International	1560604X (Print)	Active	2002-ongoing	N/A	European Association of Thermology	278	N/A
89	Journal of the Australian Traditional-Medicine Society	13263390 (Print)	Inactive	2008-2016	N/A	Australian Traditional-Medicine Society	276	N/A (Inactive)
90	Journal of Traditional Medicines	18801447 (Print); 18813747 (Electronic)	Inactive	2004-2013	N/A	Medical and Pharmaceutical Society for WAKAN-YAKU	268	N/A (Inactive)
91	Arztezeitschrift für Naturheilverfahren und Regulationsmedizin	16148339 (Print)	Inactive	2004-2006	Continued as KIM - Komplementare und Integrative Medizin, Artztezeitschrift fur Naturheilverfahren see position #81	Medizinisch Literarische Verlagsgesellschaft mbH	266	N/A (Inactive)
92	Revista Medica de Homeopatia	18888526 (Print)	Inactive	2008-2017	N/A	Elsevier Doyma	261	N/A (Inactive)
93	Pharmacognosy Reviews	09737847 (Print); 09762787 (Electronic)	Inactive	2009-2018 (cancelled)	N/A	Medknow Publications	255	N/A (Inactive)
94	Advances in Integrative Medicine	22129588 (Print); 22129596 (Electronic)	Active	2013-ongoing	N/A	Elsevier BV	254	N/A
95	Journal of Chiropractic Humanities	15563499 (Print)	Active	2010-ongoing	N/A	Elsevier BV	247	N/A
96	Asian Medicine	1573420X (Print); 15734218 (Electronic)	Active	2007-ongoing	N/A	Brill	241	N/A
97	Natural Solutions	19408153 (Print)	Inactive	2009-2011, 1996-1997	N/A	Alternative Medicine.com	226	N/A (Inactive)
98	Spirituality in Clinical Practice	23264500 (Print); 23264519 (Electronic)	Active	2014-ongoing	N/A	American Psychological Association Inc.	222	N/A
99	Alternative Medicine Alert	1096942X (Print)	Inactive	2009-2012	Continued as Integrative Medicine Alert; see position #78	American Health Consultants, Inc.	220	N/A (Inactive)
100	Journal of Complementary Medicine	14468263 (Print)	Inactive	2008-2009	N/A	Australian Pharmaceutical Publishing Co., Ltd.	205	N/A (Inactive)
101	World Journal of Traditional Chinese Medicine	23118571 (Print); 25892894 (Electronic)	Active	2017-ongoing	N/A	Wolters Kluwer Medknow Publications	200	N/A
102	Australian Journal of Medical Herbalism	10338330 (Print)	Inactive	2006-2017	Continued as Australian Journal of Herbal and Naturopathic Medicine; see position #130	National Herbalists Association of Australia	177	N/A (Inactive)
103	International Journal of Applied Research in Natural Products	19406223 (Print)	Inactive	2008-2016 (cancelled)	N/A	Healthy Synergies Publications	176	N/A (Inactive)
104	Nordisk Tidsskrift for Musikkterapi	08039828 (Print)	Inactive	1992-2000	Continued as Nordic Journal of Music Therapy; see position #67	Taylor and Francis Ltd.	175	N/A (Inactive)
105	Journal of Pharmacopuncture	20936966 (Print); 22346856 (Electronic)	Active	2016-ongoing	N/A	Korean Pharmacopuncture Institute	173	N/A
106	European Journal of Oriental Medicine	13516647 (Print)	Inactive	2017, 2006-2014	N/A	British Acupuncture Council	159	N/A (Inactive)
107	Phytomedica	09723293 (Print)	Inactive	1999-2006	Formerly known as Indian Journal of Indigenous Medicines; not categorized as “complementary and alternative medicine” by Scopus	Scientific Publishers	158	N/A (Inactive)
108	Advances in Traditional Medicine	26624052 (Print); 26624060 (Electronic)	Active	2020-ongoing	Formerly known as Oriental Pharmacy and Experimental Medicine; see position #80	Springer Singapore	157	N/A
109	Chiropractic Journal of Australia	10360913 (Print)	Active	2016-ongoing, 2010-2013	N/A	Chiropractors' Association of Australia	151	N/A
110	Journal of Sports Chiropractic and Rehabilitation	10841288 (Print)	Inactive	1996-2001	Formerly known as Chiropractic Sports Medicine; see position #115	Atwood Publishing	147	N/A (Inactive)
111	Cannabis and Cannabinoid Research	23788763 (Electronic)	Active	2016-ongoing	N/A	Mary Ann Liebert Inc.	147	N/A
112	Revista de Fitoterapia	15760952 (Print); 19885806 (Electronic)	Active	2009-ongoing	N/A	Cita Publicaciones y Documentacion SL	144	N/A
113	Chiropractic and Osteopathy	17461340 (Print)	Inactive	2005-2010	Continued as Chiropractic and Manual Therapies; see position #59	BioMed Central	135	N/A (Inactive)
114	Scientific Review of Alternative Medicine	10950656 (Print)	Inactive	2000-2007	N/A	Prometheus Books Inc.	134	N/A (Inactive)
115	Chiropractic Sports Medicine	08896976 (Print)	Inactive	1987-1995	Continued as Journal of Sports Chiropractic and Rehabilitation; see position #110	Atwood Publishing	133	N/A (Inactive)
116	Clinical Acupuncture and Oriental Medicine	14611449 (Print)	Inactive	1999-2003	N/A	Churchill Livingstone	133	N/A (Inactive)
117	International Journal of Ozone Therapy	19723539 (Print)	Inactive	2007-2013	N/A	Centauro SRL	129	N/A (Inactive)
118	Journal of the Society for Integrative Oncology	1715894X (Print)	Inactive	2006-2010	Formerly known as Journal of Cancer Integrative Medicine; see position #140	B.C. Decker Inc.	128	N/A (Inactive)
119	Journal of Intercultural Ethnopharmacology	21468397 (Electronic)	Inactive	2016-2017 (cancelled)	N/A	Ejmanager LLC	122	N/A (Inactive)
120	Medicina Clinica e Termale	11239395 (Print)	Inactive	2008, 2005-2006, 2001-2003, 1997-1998	N/A	Tipografia la Commerciale s.n.c.	122	N/A (Inactive)
121	Phytotherapie Europeenne	16286847 (Print)	Inactive	2007-2010	N/A	Meditions Carline	121	N/A (Inactive)
122	Alternative Medicine	10814000 (Print)	Inactive	2007-2008	N/A	Future Medicine Pub.	119	N/A (Inactive)
123	Complementary Medical Research	02684055 (Print)	Inactive	1988-1992	Continued as Complementary Therapies in Medicine; see position #16	Routledge & Kegan Paul	119	N/A (Inactive)
124	Journal of Traditional Chinese Medical Sciences	20957548 (Electronic)	Active	2019-ongoing	N/A	Beijing University of Chinese Medicine	115	N/A
125	Journal of Evidence-Based Integrative Medicine	2515690X (Electronic)	Active	2018-ongoing	Formerly known as Journal of Evidence-Based Complementary and Alternative Medicine; see positions #77, 79 #125 and #136	SAGE Publications Ltd	113	N/A
126	Alternative Therapies in Womens Health	15223396 (Print)	Inactive	2006-2009	N/A	American Health Consultant	97	N/A (Inactive)
127	Integrative Medicine Research	22134220 (Print); 22134239 (Electronic)	Active	2020-ongoing	N/A	Elsevier BV	96	2.172
128	Australian Journal of Acupuncture and Chinese Medicine	18339735 (Print)	Inactive	2016-2016, 2011-2014	N/A	Australian Acupuncture and Chinese Medicine Association Ltd	83	N/A (Inactive)
129	AAO Journal	23755776 (Print)	Active	2006-ongoing	N/A	American Academy Of Osteopathy	83	N/A
130	Australian Journal of Herbal and Naturopathic Medicine	2209119X (Print); 22091203 (Electronic)	Active	2018-ongoing	Formerly known as Australian Journal of Medical Herbalism; see position #102	Naturopaths and Herbalists Association of Australia	63	N/A
131	Indian Journal of Research in Homoeopathy	09747168 (Print); 23207094 (Electronic)	Active	2019-ongoing	N/A	Wolters Kluwer Medknow Publications	61	N/A
132	Akupunktur und Traditionelle Chinesische Medizin	16146891 (Print)	Inactive	2004-2006	Formerly known as Akupunktur; not categorized as “complementary and alternative medicine” by Scopus	Medizinisch Literarische Verlagsgesellschaft mbH	59	N/A (Inactive)
133	Seminars in Preventive and Alternative Medicine	15564061 (Print)	Inactive	2005-2007	N/A	Elsevier	43	N/A (Inactive)
134	Open Access Journal of Medicinal and Aromatic Plants	09747877 (Print)	Active	2010-ongoing	N/A	Medicinal and Aromatic Plants Association of India	41	N/A
135	Archives of Drug Information	17535174 (Print)	Inactive	2008-2011	N/A	John Wiley & Sons Inc.	35	N/A (Inactive)
136	Integrative Medicine Insights	11773936 (Print)	Inactive	2008-2018	Continued as Journal of Evidence-Based Integrative Medicine; see positions #79 and #125	Libertas Academica	33	N/A (Inactive)
137	Evidence-Based Integrative Medicine	11762330 (Print)	Inactive	2005	N/A	Adis Press	31	N/A (Inactive)
138	Current Traditional Medicine	22150838 (Print); 22150846 (Electronic)	Inactive	2015-2016	N/A	Bentham Science Publishers B.V.	16	N/A (Inactive)
139	Journal of Experimental and Integrative Medicine	13094572 (Print); 21463298 (Electronic)	Inactive	2014-2016	N/A	Gesdav	16	N/A (Inactive)
140	Journal of Cancer Integrative Medicine	15446301 (Print)	Inactive	2005	Continued as Journal of the Society for Integrative Oncology; see position #118	Prime National Publishing Corp.	15	N/A (Inactive)
141	International Journal of Clinical Acupuncture	10471979 (Print)	Inactive	2014-2016	N/A	Allerton Press Inc.	4	N/A (Inactive)
142	World Journal of Acupuncture - Moxibustion	10035257 (Electronic)	Inactive	2013	N/A	Elsevier BV	4	N/A (Inactive)
143	Journal of Orthomolecular Medicine	08344825 (Print)	Inactive	2017, 1988-2015	Formerly known as Journal of Orthomolecular Psychiatry; not categorized as “complementary and alternative medicine” by Scopus	Canadian Schizophrenia Foundation	0	N/A (Inactive)

The subject area containing the largest number of publications was medicine (*n* = 172,456), followed by pharmacology, toxicology and pharmaceutics (*n* = 86,902), then biochemistry, genetics and molecular biology (*n* = 40,262). Publications were primarily published in English (*n* = 135,718), followed by Chinese (*n* = 24,614), then German (*n* = 8611). The most common document types were article (*n* = 139,540) and review (*n* = 13,418); articles primarily include original research, while reviews include literature, scoping, and systematic reviews. The most productive countries included China (*n* = 45,860), the United States (*n* = 29,523), and Germany (*n* = 10,120). The most common affiliations were the China Academy of Chinese Medical Sciences (*n* = 3560), the Beijing University of Chinese Medicine (*n* = 2896), and the Chinese Academy of Sciences (*n* = 2896); the most common funding sponsors were the National Natural Science Foundation of China (*n* = 5711), the National Institutes of Health (*n* = 4055), and the US Department of Health and Human Services (*n* = 4032). The general characteristics of eligible publications are summarized in Table 3. In addition, the 100 most highly published authors are provided in Table 4, and the 100 highest-cited publications are provided in Table 5.

**Table 3:** General Characteristics of Publications in TCAIM Journals

Publication Volume			
Number of Total Publications		*n* = 172466	100.0%
Number of Open Access Publications		*n* = 42331	24.5%
Document Type (# of publications)	Article	*n* = 139540	80.9%
	Review	*n* = 13418	7.8%
	Note	*n* = 5873	3.4%
	Editorial	*n* = 4643	2.7%
	Letter	*n* = 3096	1.8%
	Conference Paper	*n* = 2706	1.6%
	Short Survey	*n* = 1774	1.0%
	Erratum	*n* = 1293	0.7%
	Retracted	*n* = 23	0.0%
	Conference Review	*n* = 5	0.0%
	Undefined	*n* = 95	0.1%
Number of Unique Authors		*n* = 219680	
Source Titles (Journals) Across All Publications		*n* = 144	
Subject Area of Publication (10 Highest)			
(# of publications)	Medicine	*n* = 172456	100.0%
	Pharmacology, Toxicology and Pharmaceutics	*n* = 86902	50.4%
	Biochemistry, Genetics and Molecular Biology	*n* = 40262	23.3%
	Chemistry	*n* = 31845	18.5%
	Agricultural and Biological Sciences	*n* = 12124	7.0%
	Health Professions	*n* = 11487	6.7%
	Nursing	*n* = 7428	4.3%
	Social Sciences	*n* = 1949	1.1%
	Mathematics	*n* = 1461	0.8%
	Arts and Humanities	*n* = 1249	0.7%
Language of Publication (10 Highest)			
(# of publications)	English	*n* = 135718	78.7%
	Chinese	*n* = 24614	14.3%
	German	*n* = 8611	5.0%
	Spanish	*n* = 1741	1.0%
	French	*n* = 1645	1.0%
	Portuguese	*n* = 1065	0.6%
	Persian	*n* = 691	0.4%
	Polish	*n* = 200	0.1%
	Italian	*n* = 162	0.1%
	Arabic	*n* = 120	0.1%
Country of Publication (20 Highest)			
(# of publications)	China	*n* = 45860	26.6%
	United States	*n* = 29523	17.1%
	Germany	*n* = 10120	5.9%
	India	*n* = 9116	5.3%
	Japan	*n* = 6774	3.9%
	South Korea	*n* = 6120	3.5%
	United Kingdom	*n* = 6057	3.5%
	Brazil	*n* = 4915	2.8%
	Australia	*n* = 3844	2.2%
	Taiwan	*n* = 3660	2.1%
	Iran	*n* = 3558	2.1%
	Italy	*n* = 3341	1.9%
	France	*n* = 3325	1.9%
	Canada	*n* = 2607	1.5%
	Spain	*n* = 2449	1.4%
	Switzerland	*n* = 2238	1.3%
	Austria	*n* = 1835	1.1%
	Nigeria	*n* = 1814	1.1%
	Malaysia	*n* = 1798	1.0%
	Turkey	*n* = 1754	1.0%
Institutional Affiliation (20 Highest)			
(# of publications)	China Academy of Chinese Medical Sciences	*n* = 3560	2.1%
	Beijing University of Chinese Medicine	*n* = 2896	1.7%
	Chinese Academy of Sciences	*n* = 2896	1.7%
	Chinese Academy of Medical Sciences & Peking Union Medical College	*n* = 2613	1.5%
	Ministry of Education China	*n* = 2595	1.5%
	Shanghai University of Traditional Chinese Medicine	*n* = 1949	1.1%
	Nanjing University of Traditional Chinese Medicine	*n* = 1868	1.1%
	China Pharmaceutical University	*n* = 1403	0.8%
	Kyung Hee University	*n* = 1302	0.8%
	Tianjin University of Traditional Chinese Medicine	*n* = 1171	0.7%
	China Medical University Taichung	*n* = 1165	0.7%
	Chengdu University of Traditional Chinese Medicine	*n* = 1153	0.7%
	Shenyang Pharmaceutical University	*n* = 1147	0.7%
	Guangzhou University of Chinese Medicine	*n* = 1078	0.6%
	Institute of Materia Medica, Chinese Academy of Medical Sciences & Peking Union Medical College	*n* = 1069	0.6%
	Institute of Medicinal Plant Development, Chinese Academy of Medical Sciences & Peking Union Medical College	*n* = 1035	0.6%
	Peking University	*n* = 963	0.6%
	Kunming Institute of Botany Chinese Academy of Sciences	*n* = 941	0.5%
	Jiangxi University of Traditional Chinese Medicine	*n* = 853	0.5%
	Korea Institute of Oriental Medicine	*n* = 785	0.5%
Funding Sponsor (20 Highest)			
(# of publications)	National Natural Science Foundation of China	*n* = 5711	3.3%
	National Institutes of Health	*n* = 4055	2.4%
	US Department of Health and Human Services	*n* = 4032	2.3%
	National Cancer Institute	*n* = 1477	0.9%
	Ministry of Education, Culture, Sports, Science and Technology	*n* = 1057	0.6%
	Ministry of Science and Technology of the People’s Republic of China	*n* = 979	0.6%
	National Research Foundation of Korea	*n* = 944	0.5%
	National Center for Complementary and Integrative Health	*n* = 932	0.5%
	Japan Society for the Promotion of Science	*n* = 903	0.5%
	Ministry of Education of the People’s Republic of China	*n* = 749	0.4%
	Conselho Nacional de Desenvolvimento Científico e Tecnológico	*n* = 694	0.4%
	Ministério da Ciência, Tecnologia e Inovações	*n* = 574	0.3%
	Coordenação de Aperfeicoamento de Pessoal de Nível Superior	*n* = 527	0.3%
	National Institute of General Medical Sciences	*n* = 442	0.3%
	National Center for Research Resources	*n* = 427	0.3%
	National Key Research and Development Program of China	*n* = 397	0.2%
	Ministry of Science, ICT and Future Planning	*n* = 353	0.2%
	Fundamental Research Funds for the Central Universities	*n* = 323	0.2%
	European Commission	*n* = 309	0.2%
	Chinese Academy of Sciences	*n* = 293	0.2%

**Table 4:** 100 Most Productive Authors Across Publications in TCAIM Journals

Position	Author Name	Number of Publications	Author H-Index
1	Huang, L.Q.	330	42
2	Goetz, P.	314	5
3	Ernst, E.	311	106
4	Xie, Y.M.	264	14
5	Lee, M.S.	232	47
6	Tu, P.F.	231	48
7	Pezzuto, J.M.	223	88
8	Jia, X.B.	213	33
9	Kinghorn, A.D.	207	72
10	Cordell, G.A.	205	61
11	Guo, L.P.	186	23
12	Yang, M.	186	18
13	Dossey, L.	179	12
14	Wang, Z.Z.	176	27
15	Kingston, D.G.I.	170	60
16	Yao, X.S.	169	51
17	Khan, I.A.	168	60
18	Farnsworth, N.R.	167	66
19	White, A.	167	56
20	Hostettmann, K.	166	63
21	Schulz, V.	162	9
22	Hamburger, M.	161	46
23	Qin, X.M.	161	27
24	Allen, T.W.	160	4
25	Donnelly, G.F.	160	5
26	Sun, H.D.	156	50
27	Duan, J.A.	153	44
28	Proksch, P.	152	65
29	Wu, Y.C.	152	56
30	Guo, Q.S.	151	18
31	Setzer, W.N.	146	43
32	Adams, J.	145	38
33	Efferth, T.	145	75
34	Ammer, K.	140	16
35	Ye, W.C.	140	42
36	Zhang, T.J.	140	15
37	Bauer, R.	137	51
38	Yang, S.L.	136	26
39	Hart, J.	134	5
40	Lee, K.H.	134	80
41	Sticher, O.	134	50
42	Yarnell, E.	134	13
43	Pettit, G.R.	133	89
44	Uehleke, B.	133	16
45	Yang, X.W.	131	31
46	Chen, S.L.	130	57
47	Wang, Z.M.	128	18
48	Xiao, X.H.	127	38
49	Lin, J.G.	126	46
50	Beyer, L.	124	6
51	Kiefer, D.	124	7
52	Choudhary, M.I.	122	57
53	Pieters, L.	122	58
54	Chen, R.Y.	120	57
55	Robinson, N.	120	33
56	Verpoorte, R.	119	86
57	Liao, X.	118	11
58	Zhang, W.D.	116	46
59	Hao, X.J.	115	42
60	Block, K.I.	114	17
61	Kraft, K.	114	9
62	Liebenson, C.	114	9
63	Kadota, S.	112	66
64	Lao, L.	112	50
65	Litscher, G.	112	29
66	Wagner, H.	112	49
67	Niemtzow, R.C.	111	14
68	Yu, D.Q.	111	28
69	Fisher, P.	110	26
70	Kong, L.Y.	110	47
71	Omura, Y.	110	17
72	McLaughlin, J.L.	108	55
73	Tezuka, Y.	108	61
74	Chang, F.R.	107	54
75	Fong, H.H.S.	106	55
76	Gibson, D.M.	106	1
77	Cramer, H.	105	41
78	Yuan, Y.	105	16
79	Horowitz, S.	104	7
80	Walach, H.	104	40
81	Gerwick, W.H.	103	73
82	Wright, A.D.	103	49
83	Naghdi Badi, H.	102	18
84	Qian, D.W.	102	31
85	Kuo, Y.H.	101	53
86	Schwartz, S.A.	101	7
87	Dai, H.F.	100	31
88	Guo, D.A.	100	56
89	Hsieh, C.L.	100	36
90	Xiao, P.G.	100	42
91	Li, P.	99	56
92	Morita, H.	99	52
93	De Tommasi, N.	98	39
94	Ots, T.	98	4
95	Tokuda, H.	97	68
96	Weeks, J.	97	8
97	Witt, C.M.	97	42
98	Saller, R.	96	33
99	Steel, A.	96	22
100	König, G.M.	95 (tied for 100th place)	54
101	Xiao, W.	95 (tied for 100th place)	14

**Table 5:** 100 Highest-Cited Publications in TCAIM Journals

Position	Title	Authors	Year	Source Title	Citation Count
1	Natural products as sources of new drugs over the last 25 years	Newman D.J., Cragg G.M.	2007	Journal of Natural Products	3258
2	Flavonoids as antioxidants	Pietta P.-G.	2000	Journal of Natural Products	3162
3	Natural products as sources of new drugs over the 30 years from 1981 to 2010	Newman D.J., Cragg G.M.	2012	Journal of Natural Products	3122
4	Brine shrimp: A convenient general bioassay for active plant constituents	Meyer B.N., Ferrigni N.R., Putnam J.E., Jacobsen L.B., Nichols D.E., McLaughlin J.L.	1982	Planta Medica	2939
5	Natural products as sources of new drugs from 1981 to 2014	Newman D.J., Cragg G.M.	2016	Journal of Natural Products	2750
6	Natural products as sources of new drugs over the period 1981–2002	Newman D.J., Cragg G.M., Snader K.M.	2003	Journal of Natural Products	2285
7	Free radicals, antioxidants and functional foods: Impact on human health	Lobo V., Patil A., Phatak A., Chandra N.	2010	Pharmacognosy Reviews	1939
8	Pharmacology of *Curcuma longa*	Ammon H.P.T., Wahl M.A.	1991	Planta Medica	1415
9	A sensitive and quick microplate method to determine the minimal inhibitory concentration of plant extracts for bacteria	Eloff J.N.	1998	Planta Medica	1320
10	Screening of plant extracts for antioxidant activity: A comparative study on three testing methods	Koleva I.I., Van Beek T.A., Linssen J.P.H., De Groot A., Evstatieva L.N.	2002	Phytochemical Analysis	1186
11	Influence of piperine on the pharmacokinetics of curcumin in animals and human volunteers	Shoba G., Joy D., Joseph T., Majeed M., Rajendran R., Srinivas P.S.S.R.	1998	Planta Medica	1178
12	Natural products in drug discovery and development	Cragg G.M., Newman D.J., Snader K.M.	1997	Journal of Natural Products	1126
13	Natural polyphenols (vegetable tannins) as drugs: Possible modes of action	Haslam E.	1996	Journal of Natural Products	960
14	Preferred reporting items for systematic reviews and meta-analyses: The PRISMA statement (Chinese edition)	Moher D., Liberati A., Tetzlaff J., Altman D.G., Altman D., Antes G., Atkins D., Barbour V., Barrowman N., Berlin J.A., Clark J., Clarke M., Cook D., D’Amico R., Deeks J.J., Devereaux P.J., Dickersin K., Egger M., Ernst E., Gøtzsche P.C., Grimshaw J., Guyatt G., Higgins J., Ioannidis J.P.A., Kleijnen J., Lang T., Magrini N., McNamee D., Moja L., Mulrow C., Napoli M., Oxman A., Pham B., Rennie D., Sampson M., Schulz K.F., Shekelle P.G., Tovey D., Tugwell P.	2009	Journal of Chinese Integrative Medicine	933
15	The role of natural product chemistry in drug discovery	Butler M.S.	2004	Journal of Natural Products	918
16	Natural products from endophytic microorganisms	Strobel G., Daisy B., Castillo U., Harper J.	2004	Journal of Natural Products	916
17	Anti-inflammatory properties of curcumin, a major constituent of *Curcuma longa*: A review of preclinical and clinical research	Jurenka J.S.	2009	Alternative Medicine Review	837
18	Dose escalation of a curcuminoid formulation	Lao C.D., Ruffin IV M.T., Normolle D., Heath D.D., Murray S.I., Bailey J.M., Boggs M.E., Crowell J., Rock C.L., Brenner D.E.	2006	BMC Complementary and Alternative Medicine	833
19	Structure-activity relationship and classification of flavonoids as inhibitors of xanthine oxidase and superoxide scavengers	Cos P., Ying L., Calomme M., Hu J.P., Cimanga K., Van Poel B., Pieters L., Vlietinck A.J., Vanden Berghe D.	1998	Journal of Natural Products	823
20	Mindfulness-based stress reduction for stress management in healthy people: A review and meta-analysis	Chiesa A., Serretti A.	2009	Journal of Alternative and Complementary Medicine	789
21	Th1/Th2 balance: The hypothesis, its limitations, and implications for health and disease	Kidd P.	2003	Alternative Medicine Review	781
22	Annonaceous acetogenins: Recent progress	Alali F.Q., Liu X.-X., McLaughlin J.L.	1999	Journal of Natural Products	745
23	Antidiabetic plants and their active constituents	Marles R.J., Farnsworth N.R.	1995	Phytomedicine	744
24	Trends in use of complementary and alternative medicine by us adults: 1997–2002	Tindle H.A., Davis R.B., Phillips R.S., Eisenberg D.M.	2005	Alternative Therapies in Health and Medicine	724
25	Antioxidant principles from Bauhinia tarapotensis	Braca A., De Tommasi N., Di Bari L., Pizza C., Politi M., Morelli I.	2001	Journal of Natural Products	712
26	Synergy research: Approaching a new generation of phytopharmaceuticals	Wagner H., Ulrich-Merzenich G.	2009	Phytomedicine	695
27	Safety and anti-inflammatory activity of curcumin: A component of tumeric (*Curcuma longa*)	Chainani-Wu N.	2003	Journal of Alternative and Complementary Medicine	683
28	Natural products from plant-associated microorganisms: Distribution, structural diversity, bioactivity, and implications of their occurrence	Gunatilaka A.A.L.	2006	Journal of Natural Products	664
29	The pharmacological potential of mushrooms	Lindequist U., Niedermeyer T.H.J., Jülich W.-D.	2005	Evidence-based Complementary and Alternative Medicine	648
30	Fixed oil of *Nigella sativa* and derived thymoquinone inhibit eicosanoid generation in leukocytes and membrane lipid peroxidation	Houghton P.J., Zarka R., De Las Heras B., Hoult J.R.S.	1995	Planta Medica	641
31	Marine natural products and related compounds in clinical and advanced preclinical trials	Newman D.J., Cragg G.M.	2004	Journal of Natural Products	626
32	Synergy and other interactions in phytomedicines	Williamson E.M.	2001	Phytomedicine	621
33	Nrf2 as a master redox switch in turning on the cellular signaling involved in the induction of cytoprotective genes by some chemopreventive phytochemicals	Surh Y.-J., Kundu J.K., Na H.-K.	2008	Planta Medica	593
34	Use and expenditure on complementary medicine in England: A population based survey	Thomas K.J., Nicholl J.P., Coleman P.	2001	Complementary Therapies in Medicine	577
35	In vitro antibacterial activity of some plant essential oils	Prabuseenivasan S., Jayakumar M., Ignacimuthu S.	2006	BMC Complementary and Alternative Medicine	572
36	Antioxidant and antiinflammatory activities of anthocyanins and their aglycon, cyanidin, from tart cherries	Wang H., Nair M.G., Strasburg G.M., Chang Y.-C., Booren A.M., Gray J.I., DeWitt D.L.	1999	Journal of Natural Products	572
37	Alkaloids from amphibian skin: A tabulation of over eight-hundred compounds	Daly J.W., Spande T.F., Garraffo H.M.	2005	Journal of Natural Products	558
38	Scientific basis for the therapeutic use of *Withania somnifera* (ashwagandha): A review	Mishra L.-C., Singh B.B., Dagenais S.	2000	Alternative Medicine Review	538
39	Acetylcholinesterase inhibitors from plants	Mukherjee P.K., Kumar V., Mal M., Houghton P.J.	2007	Phytomedicine	522
40	Extraction, isolation and characterization of bioactive compounds from plants’ extracts	Sasidharan S., Chen Y., Saravanan D., Sundram K.M., Yoga Latha L.	2011	African Journal of Traditional, Complementary and Alternative Medicines	514
41	Annonaceous acetogenins: A review	Rupprecht J.K., Hui Y.-H., McLaughlin J.L.	1990	Journal of Natural Products	498
42	Synergism between natural products and antibiotics against infectious diseases	Hemaiswarya S., Kruthiventi A.K., Doble M.	2008	Phytomedicine	489
43	Lead toxicity, a review of the literature. Part I: Exposure, evaluation, and treatment	Patrick L.	2006	Alternative Medicine Review	481
44	Complementary and alternative medicine use in Australia: A national population-based survey	Xue C.C.L., Zhang A.L., Lin V., Da Costa C., Story D.F.	2007	Journal of Alternative and Complementary Medicine	460
45	Traditional Chinese medicine network pharmacology: Theory, methodology and application	Li S., Zhang B.	2013	Chinese Journal of Natural Medicines	455
46	Beneficial effects of green tea: A literature review	Chacko S.M., Thambi P.T., Kuttan R., Nishigaki I.	2010	Chinese Medicine	454
47	Therapeutic applications of pomegranate (*Punica granatum* L.): A review	Jurenka J.	2008	Alternative Medicine Review	451
48	Recent extraction techniques for natural products: Microwave-assisted extraction and pressurised solvent extraction	Kaufmann B., Christen P.	2002	Phytochemical Analysis	447
49	Astaxanthin, a carotenoid with potential in human health and nutrition	Hussein G., Sankawa U., Goto H., Matsumoto K., Watanabe H.	2006	Journal of Natural Products	429
50	Antimicrobial activity of essential oils: A 1976–1986 literature review. Aspects of the test methods	Janssen A.M., Scheffer J.J.C., Baerheim Svendsen A.	1987	Planta Medica	429
51	Antioxidant activity, total phenolic and total flavonoid contents of whole plant extracts *Torilis leptophylla* L	Saeed N., Khan M.R., Shabbir M.	2012	BMC Complementary and Alternative Medicine	426
52	Clinical applications of N-acetylcysteine	Kelly G.S.	1998	Alternative Medicine Review	422
53	Nutritional quality of organic versus conventional fruits, vegetables, and grains	Worthington V.	2001	Journal of Alternative and Complementary Medicine	421
54	Chemotherapy-associated oxidative stress: Impact on chemotherapeutic effectiveness	Conklin K.A.	2004	Integrative Cancer Therapies	419
55	Iridoids. A review	El-Naggar L.J., Beal J.L.	1980	Journal of Natural Products	418
56	Anti-aids agents, 11. Betulinic acid and platanic acid as anti-HIV principles from *Syzigium claviflorum*, and the anti-HIV activity of structurally related triterpenoids	Fujioka T., Kashiwada Y., Kilkuskie R.E., Cosentino L.M., Bailas L.M., Jiang J.B., Janzen W.P., Chen I.-S., Lee K.-H.	1994	Journal of Natural Products	415
57	Bleomycin: New perspectives on the mechanism of action	Hecht S.M.	2000	Journal of Natural Products	406
58	Plant-derived leading compounds for chemotherapy of human immunodeficiency virus (HIV) infection	Vlietinck A.J., De Bruyne T., Apers S., Pieters L.A.	1998	Planta Medica	404
59	Antioxidants and cancer III: Quercetin	Lamson D.W., Brignall M.S.	2000	Alternative Medicine Review	403
60	A microwell cytotoxicity assay using *Artemia salina* (brine shrimp)	Solis P.N., Wright C.W., Anderson M.M., Gupta M.P., Phillipson J.D.	1993	Planta Medica	403
61	How many cancer patients use complementary and alternative medicine: A systematic review and metaanalysis	Horneber M., Bueschel G., Dennert G., Less D., Ritter E., Zwahlen M.	2012	Integrative Cancer Therapies	395
62	The BBC survey of complementary medicine use in the UK	Ernst E., White A.	2000	Complementary Therapies in Medicine	395
63	The scientific rediscovery of an ancient Chinese herbal medicine: *Cordyceps sinensis* part I	Zhu J.-S., Halpern G.M., Jones K.	1998	Journal of Alternative and Complementary Medicine	389
64	Therapeutic applications of whey protein	Marshall K.	2004	Alternative Medicine Review	383
65	Chemical toxins: A hypothesis to explain the global obesity epidemic	Baillie-Hamilton P.F.	2002	Journal of Alternative and Complementary Medicine	377
66	Quantitative 1H NMR: Development and potential of a method for natural products analysis	Pauli G.F., Jaki B.U., Lankin D.C.	2005	Journal of Natural Products	376
67	Antimicrobial activity of some ethnomedicinal plants used by Paliyar tribe from Tamil Nadu, India	Duraipandiyan V., Ayyanar M., Ignacimuthu S.	2006	BMC Complementary and Alternative Medicine	375
68	Goji (*Lycium barbarum* and *L. chinense*): Phytochemistry, pharmacology and safety in the perspective of traditional uses and recent popularity	Potterat O.	2010	Planta Medica	374
69	Constituents of *Cannabis sativa* *L*. XVII. a review of the natural constituents	Turner C.E., Elsohly M.A., Boeren E.G.	1980	Journal of Natural Products	373
70	Recent natural products based drug development: A pharmaceutical industry perspective	Shu Y.-Z.	1998	Journal of Natural Products	371
71	Cyclooxygenase inhibitory and antioxidant cyanidin glycosides in cherries and berries	Seeram N.P., Momin R.A., Nair M.G., Bourquin L.D.	2001	Phytomedicine	370
72	*Zingiberis rhizoma*: A comprehensive review on the ginger effect and efficacy profiles	Chrubasik S., Pittler M.H., Roufogalis B.D.	2005	Phytomedicine	368
73	Ayurveda and traditional Chinese medicine: A comparative overview	Patwardhan B., Warude D., Pushpangadan P., Bhatt N.	2005	Evidence-based Complementary and Alternative Medicine	366
74	A-type proanthocyanidin trimers from cranberry that inhibit adherence of uropathogenic P-fimbriated *Escherichia coli*	Foo L.Y., Lu Y., Howell A.B., Vorsa N.	2000	Journal of Natural Products	366
75	Historical review of medicinal plants’ usage	Petrovska B.B.	2012	Pharmacognosy Reviews	363
76	Plant sources of hepatotoxic pyrrolizidine alkaloids	Smith L.W., Culvenor C.C.J.	1981	Journal of Natural Products	361
77	Steaming of ginseng at high temperature enhances biological activity	Wang Yu Kim, Jong Moon Kim, Sang Beom Han, Seung Ki Lee, Nak Doo Kim, Park M.K., Chong Kook Kim, Park J.H.	2000	Journal of Natural Products	360
78	Recent trends and important developments in propolis research	Bankova V.	2005	Evidence-based Complementary and Alternative Medicine	357
79	Therapeutic applications of fenugreek	Basch E., Ulbricht C., Kuo G., Szapary P., Smith M.	2003	Alternative Medicine Review	357
80	Antioxidant and free radical scavenging activity of *Spondias pinnata*	Hazra B., Biswas S., Mandal N.	2008	BMC Complementary and Alternative Medicine	354
81	Antioxidant and radical scavenging effects of aged garlic extract and its constituents	Imai J., Ide N., Nagae S., Moriguchi T., Matsuura H., Itakura Y.	1994	Planta Medica	350
82	Pentacyclic triterpenes of the lupane, oleanane and ursane group as tools in cancer therapy	Laszczyk M.N.	2009	Planta Medica	346
83	Antioxidant and antimicrobial activity of *Foeniculum vulgare* and crithmum maritimum essential oils	Ruberto G., Baratta M.T., Deans S.G., Dorman H.J.D.	2000	Planta Medica	346
84	Medicinal plants used by traditional healers in Kancheepuram District of Tamil Nadu, India	Muthu C., Ayyanar M., Raja N., Ignacimuthu S.	2006	Journal of Ethnobiology and Ethnomedicine	344
85	Lead toxicity part II: The role of free radical damage and the use of antioxidants in the pathology and treatment of lead toxicity	Patrick L.	2006	Alternative Medicine Review	343
86	A rapid and effective method for RNA extraction from different tissues of grapevine and other woody plants	Gambino G., Perrone I., Gribaudo I.	2008	Phytochemical Analysis	341
87	Benefits and requirements of vitamin D for optimal health: A review	Grant W.B., Holick M.F.	2005	Alternative Medicine Review	341
88	Antimicrobial and antioxidant activities of three Mentha species essential oils	Mimica-Dukić N., Božin B., Soković M., Mihajlović B., Matavulj M.	2003	Planta Medica	338
89	The taxane diterpenoids	Baloglu E., Kingston D.G.I.	1999	Journal of Natural Products	337
90	Flavonoids: A versatile source of anticancer drugs	Chahar M.K., Sharma N., Dobhal M.P., Joshi Y.C.	2011	Pharmacognosy Reviews	332
91	Resilience: A historical review of the construct	Tusaie K., Dyer J.	2004	Holistic Nursing Practice	332
92	α-glucosidase inhibitors from plants: A natural approach to treat diabetes	Kumar S., Narwal S., Kumar V., Prakash O.	2011	Pharmacognosy Reviews	331
93	Flavonoids from *Hypericum perforatum* show antidepressant activity in the forced swimming test	Butterweck V., Jürgenliemk G., Nahrstedt A., Winterhoff H.	2000	Planta Medica	328
94	Stigmasterols from *Typha latifolia*	Greca M.D., Monaco P., Previtera L.	1990	Journal of Natural Products	327
95	Flavonoids and phenolic acids: Role and biochemical activity in plants and human	Ghasemzadeh A., Ghasemzadeh N.	2011	Journal of Medicinal Plant Research	326
96	Alternative antimicrobial approach: Nano-antimicrobial materials	Beyth N., Houri-Haddad Y., Domb A., Khan W., Hazan R.	2015	Evidence-based Complementary and Alternative Medicine	324
97	Anti-inflammatory activity of linalool and linalyl acetate constituents of essential oils	Peana A.T., D’Aquila P.S., Panin F., Serra G., Pippia P., Moretti M.D.L.	2002	Phytomedicine	324
98	Immunostimulant agents from *Andrographis paniculata*	Puri A., Saxena R., Saxena R.P., Saxena K.C., Srivastava V., Tandon J.S.	1993	Journal of Natural Products	324
99	The health benefits of yoga and exercise: A review of comparison studies	Ross A., Thomas S.	2010	Journal of Alternative and Complementary Medicine	320
100	Anti-inflammatory compounds of plant origin. Part II. Modulation of pro-inflammatory cytokines, chemokines and adhesion molecules	Calixto J.B., Campos M.M., Otuki M.F., Santos A.R.S.	2004	Planta Medica	319

**Fig. 1:**
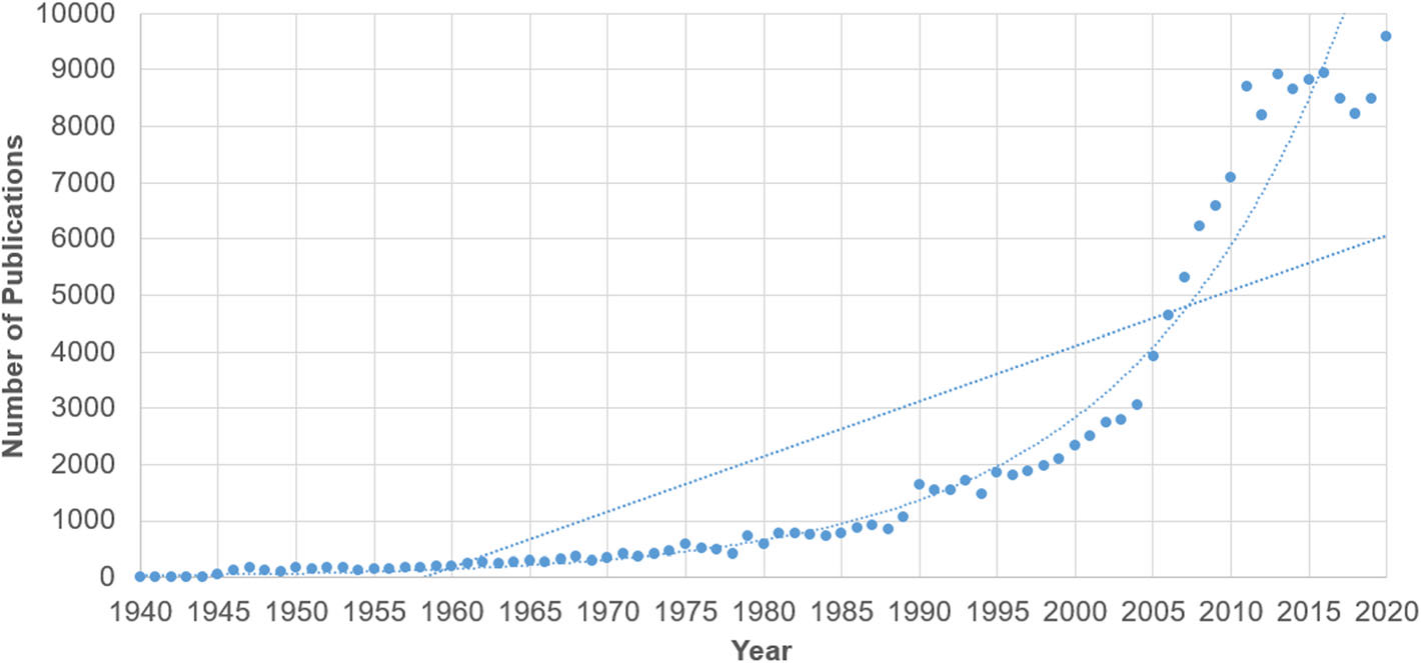
Number of Publications in Scopus-Indexed TCAIM Journals per Year from 1938 to 2020

Figure 1 depicts the number of publications published per year from 1938 to 2020, inclusive of an exponential and linear curve. Mathematical adjustment to an exponential curve (y = 30.699e^0.073x^), as shown in this figure, resulted in a correlation coefficient *r* = 0.9698, which indicates that 5.94% of variability remains unexplained by this adjustment. In contrast, the linear adjustment (y = 97.915x - 1971.9) of the measured values provides an *r* = 0.8160, and thus an unexplained variability of 33.42%. These results suggest fulfilment of Price’s Law, with scientific production within CAIM journals showing exponential growth. Additionally, the relative growth rate was found to range from 0.05 to 0.67. Doubling time was found to range from 1.04 to 15.02. Table 6 provides annual relative growth rates and doubling times.

**Table 6:** Relative Growth Rates and Doubling Times

Year	Number of Publications	Cumulative Total	W1	W2	Relative Growth Rate	Doubling Time
1938	13	13	–	2.57	–	–
1939	10	23	2.57	3.14	0.57	1.21
1940	12	35	3.14	3.56	0.42	1.65
1941	14	49	3.56	3.89	0.34	2.06
1942	12	61	3.89	4.11	0.22	3.16
1943	16	77	4.11	4.34	0.23	2.97
1944	12	89	4.34	4.49	0.14	4.78
1945	52	141	4.49	4.95	0.46	1.51
1946	134	275	4.95	5.62	0.67	1.04
1947	162	437	5.62	6.08	0.46	1.50
1948	125	562	6.08	6.33	0.25	2.75
1949	93	655	6.33	6.49	0.15	4.52
1950	170	825	6.49	6.72	0.23	3.00
1951	153	978	6.72	6.89	0.17	4.07
1952	167	1145	6.89	7.04	0.16	4.40
1953	164	1309	7.04	7.18	0.13	5.18
1954	132	1441	7.18	7.27	0.10	7.21
1955	142	1583	7.27	7.37	0.09	7.37
1956	159	1742	7.37	7.46	0.10	7.24
1957	181	1923	7.46	7.56	0.10	7.01
1958	180	2103	7.56	7.65	0.09	7.74
1959	190	2293	7.65	7.74	0.09	8.01
1960	197	2490	7.74	7.82	0.08	8.41
1961	248	2738	7.82	7.92	0.09	7.30
1962	274	3012	7.92	8.01	0.10	7.27
1963	251	3263	8.01	8.09	0.08	8.66
1964	279	3542	8.09	8.17	0.08	8.45
1965	286	3828	8.17	8.25	0.08	8.92
1966	263	4091	8.25	8.32	0.07	10.43
1967	310	4401	8.32	8.39	0.07	9.49
1968	369	4770	8.39	8.47	0.08	8.61
1969	284	5054	8.47	8.53	0.06	11.98
1970	340	5394	8.53	8.59	0.07	10.64
1971	423	5817	8.59	8.67	0.08	9.18
1972	362	6179	8.67	8.73	0.06	11.48
1973	408	6587	8.73	8.79	0.06	10.84
1974	469	7056	8.79	8.86	0.07	10.07
1975	576	7632	8.86	8.94	0.08	8.83
1976	520	8152	8.94	9.01	0.07	10.51
1977	492	8644	9.01	9.07	0.06	11.82
1978	408	9052	9.07	9.11	0.05	15.02
1979	734	9786	9.11	9.19	0.08	8.89
1980	591	10377	9.19	9.25	0.06	11.82
1981	776	11153	9.25	9.32	0.07	9.61
1982	785	11938	9.32	9.39	0.07	10.19
1983	746	12684	9.39	9.45	0.06	11.43
1984	727	13411	9.45	9.50	0.06	12.43
1985	774	14185	9.50	9.56	0.06	12.35
1986	870	15055	9.56	9.62	0.06	11.64
1987	923	15978	9.62	9.68	0.06	11.65
1988	846	16824	9.68	9.73	0.05	13.43
1989	1060	17884	9.73	9.79	0.06	11.34
1990	1628	19512	9.79	9.88	0.09	7.95
1991	1544	21056	9.88	9.96	0.08	9.10
1992	1545	22601	9.96	10.03	0.07	9.79
1993	1720	24321	10.03	10.10	0.07	9.45
1994	1475	25796	10.10	10.16	0.06	11.77
1995	1865	27661	10.16	10.23	0.07	9.93
1996	1800	29461	10.23	10.29	0.06	10.99
1997	1877	31338	10.29	10.35	0.06	11.22
1998	1982	33320	10.35	10.41	0.06	11.30
1999	2089	35409	10.41	10.48	0.06	11.40
2000	2330	37739	10.48	10.54	0.06	10.87
2001	2499	40238	10.54	10.60	0.06	10.81
2002	2751	42989	10.60	10.67	0.07	10.48
2003	2802	45791	10.67	10.73	0.06	10.97
2004	3061	48852	10.73	10.80	0.06	10.71
2005	3908	52760	10.80	10.87	0.08	9.00
2006	4637	57397	10.87	10.96	0.08	8.23
2007	5310	62707	10.96	11.05	0.09	7.83
2008	6216	68923	11.05	11.14	0.09	7.33
2009	6582	75505	11.14	11.23	0.09	7.60
2010	7088	82593	11.23	11.32	0.09	7.72
2011	8691	91284	11.32	11.42	0.10	6.93
2012	8194	99478	11.42	11.51	0.09	8.06
2013	8920	108398	11.51	11.59	0.09	8.07
2014	8642	117040	11.59	11.67	0.08	9.03
2015	8825	125865	11.67	11.74	0.07	9.53
2016	8930	134795	11.74	11.81	0.07	10.11
2017	8471	143266	11.81	11.87	0.06	11.37
2018	8212	151478	11.87	11.93	0.06	12.43
2019	8471	159949	11.93	11.98	0.05	12.73
2020	9591	169540	11.98	12.04	0.06	11.90

**Fig. 2:**
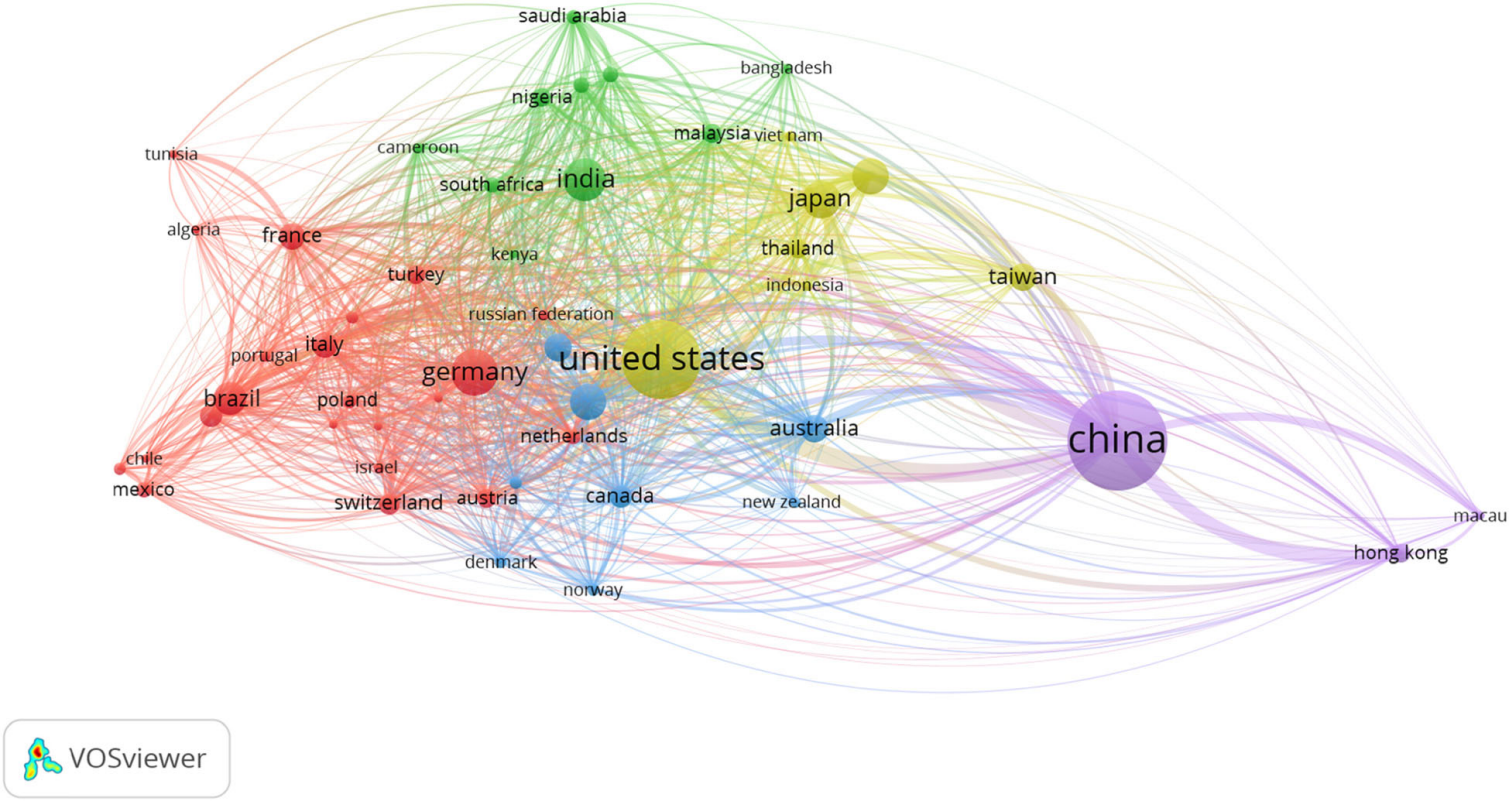
Co-Authorship Analysis of the 50 Most Productive Countries

**Fig. 3:**
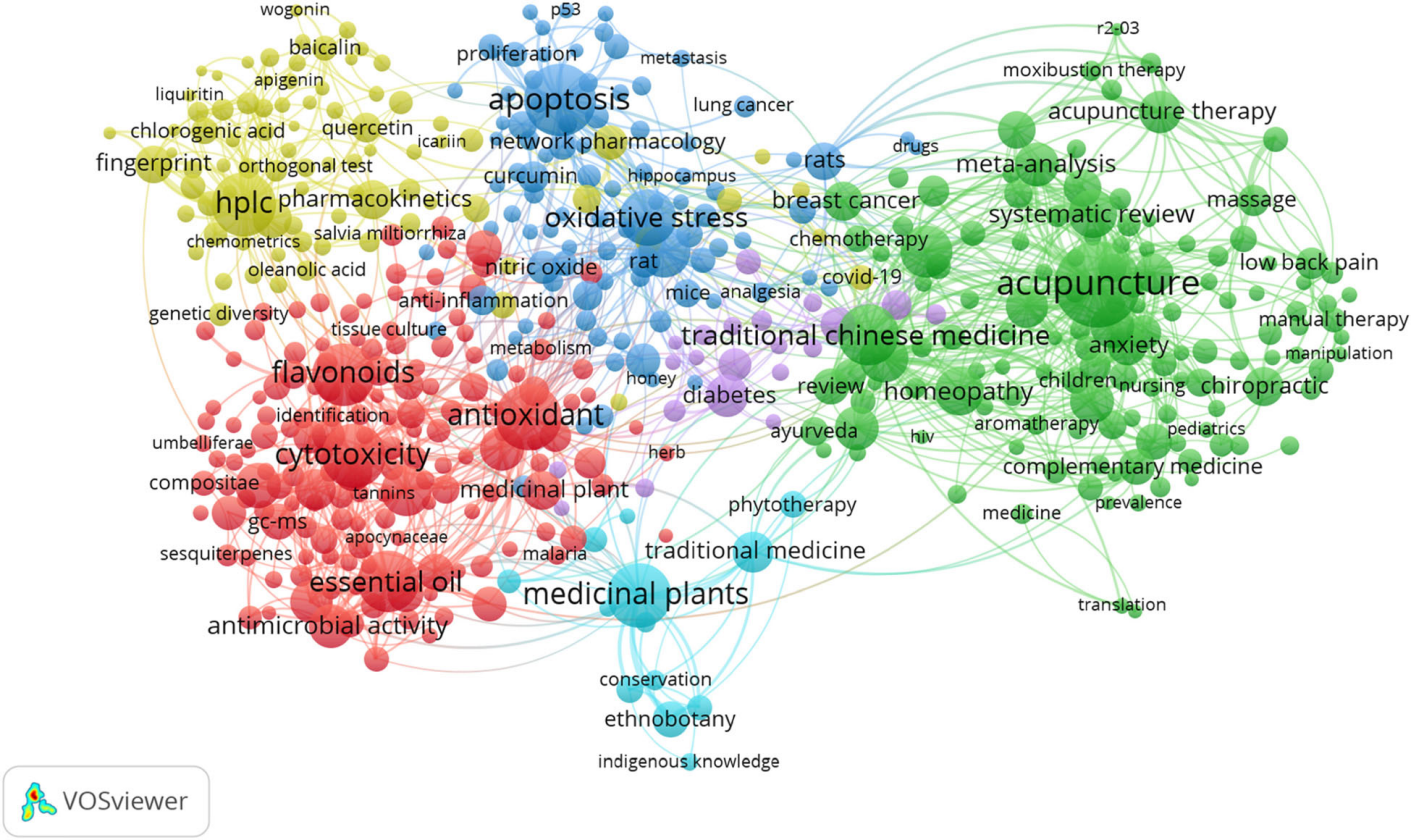
Co-Occurrence Analysis of the 500 Most Frequent Author Keywords

Bibliometric networks were constructed and visualized using the software tool VOSviewer, and include all 172,466 captured by the present study’s search. This added layer of analysis of the most influential subset of publications captured provides a greater understanding of the relationship that exists between certain items (i.e. countries, keywords, authors, journals, etc.). In each bibliometric network (figure), each item is represented in a network visualisation by a label and a circle; the weight of an item determines the size of the label and the circle of an item. Figure 2 depicts a co-authorship analysis of the 50 most productive countries. In a co-authorship analysis, the relatedness of items is determined based on the number of co-authored publications. From this figure, it can be seen that while China is the most productive country, Chinese authors tend to collaborate less with researchers in other countries as shown by the distance between lines. In contrast, American authors tend to collaborate with many countries internationally, while German authors tend to collaborate more with researchers in other European countries. Figure 3 depicts a co-occurrence analysis of the 500 most frequent author keywords used across all publications. In a co-occurrence analysis, the relatedness of items is determined based on the number of publications in which they occur together. From this figure, a number of clusters can be observed representing different TCAIM topics. The yellow, red and dark blue clusters represent a large network of keywords related to laboratory-based studies, while the green cluster represents keywords related to clinical research and review-type studies. The smaller light blue cluster also highlights research conducted on traditional and indigenous medicines. This figure also provides insights into some of the most highly studied diseases/conditions published in TCAIM journals, which include breast and lung cancer, diabetes, anxiety, and low back pain.

## Discussion

The objective of the present bibliometric analysis is to capture the characteristics of the research literature published in TCAIM journals. The search conducted on Scopus yielded over 170,000 publications, representing the largest bibliometric analysis of TCAIM literature to date to the author’s knowledge. Since the 1940s, an upward trend with respect to the volume of publications can be observed, with a steep increase observed between the mid-2000s and mid-2010s. This upward trend has continued with 2020 marking the most productive year globally to date. Unsurprisingly, therefore, the production in this body of literature follows Price’s law of exponential growth, which is characteristic of fields of research which have experienced great and continued advances and interest from the international research community; other bodies of research literature that have experienced exponential growth include the topics of medical informatics [43], glaucoma [44], psychopharmacology [45], and antipsychotic drugs [46]. This growth in the volume of research published over the most recent decades can largely be explained by an increase in funding support by government and nongovernment sectors for TCAIM research [47–50]. In the present study, it was found that China was the most productive country with respect to TCAIM research at 45,860 publications, followed by the United States at 29,523 and Germany at 10,120. A vast amount of research continues to be conducted on traditional Chinese medicine in China [51–53], while the United States and Germany have both historically been the leading countries with respect to the research of various TCAIM therapies [31–34]. While the vast majority of publications were written in English, which is largely regarded as the international language of academic publication, it is also unsurprising that the second most common language was Chinese, and the third was German, as this corresponds with the national languages of the most productive countries. Of the top 20 institutional affiliations responsible for publishing this TCAIM research, 17 originated from China, with the remaining two from South Korea and one from Taiwan; a number of affiliations based in the United States and Germany existed as well, but below the top 20. Additionally, with respect to the top 20 funding sponsors, the countries with the largest number were China and the United States, with six organizations each.

In interpreting these results, the reader should be aware of a number of caveats. For example, authors who have spent more years working in research, and journals that have been publishing for a longer period of time and/or have a greater proportion of their archive indexed in Scopus, will have more publications, citations, and collaborations. Additionally, older publications will have an increased chance of receiving citations, as evidenced by only 15 of the most 100 cited articles being published since 2020. Additionally, it is worthwhile to note that while only the journal’s impact factor was reported in Table 2, other indices are increasingly being used to rank the impact of journals (and authors), such as the H-index and SJR ranking, and differences may be observed based on the metric used.

### Comparative literature

The findings from published bibliometric analyses specific to the TCAIM research literature can be compared to that of the present study. One of the first bibliometric analyses of the TCAIM research literature was published by Barnes et al. in 1999 [31]. Using a number of TCAIM-related keywords, the authors conducted searches on MEDLINE and analysed the literature published from 1966 to 1996. At the time, they reported that the volume of TCAIM publications per year rose between 1972 and 1986, and then remained stable and approximated 1500 per year up until 1996. Although in the present study a growth in the volume of literature is still observed from 1986 to 1996, the mean number of publications per year over this decade was approximately 1400, which aligns closely with the findings of the authors [31]. Fu et al. (2011) analysed 17,002 publications found in 19 complementary and alternative medicine journals over approximately three decades [32]. They found that the most productive countries included the United States, China, India, England and Germany, all of which fell within the top seven most productive countries in the present study. A number of institutions were also identified by both Fu et al. (2011) [32] as well as the present study to be among the most productive internationally, including China Medical University and Kyung Hee University. Danell et al. analysed four decades’ worth of complementary and alternative medicine publication activity from 1966 to 2007 [33], then later repeated their study again to include five decades from 1966 to 2016 [34]. In their more recent study, they analyzed 105,216 publications, which prior to the present study, was the largest bibliometric analysis on this topic. Unlike the present study which sought to characterize publications in TCAIM journals, Danell et al.’s (2020) inclusion criteria included publications that had “Complementary Therapies” as their Medical Subject Heading major topic, in the MEDLINE database [34]. Lastly, Youn et al. (2021) conducted a bibliometric analysis of the integrative medicine research literature based on a search query using two keywords joined by the Boolean operator “OR”: “complementary and integrative medicine” OR “integrative medicine”, retrieving and analysing a total of 4660 publications. Although their study’s focus was on integrative medicine, a number of their findings are shared with the present study; for example, they also identified United States, China, and Germany to be the most productive countries (albeit in this order), and they also found that cancer was one of the most commonly studied diseases/conditions [35].

In line with the findings made by Barnes et al. (1999) [31], Danell et al. (2009) [33], Fu et al. (2011) [32], Danell et al. (2020) [34], and Youn et al. (2021) [35], the present study also found an upward trend with respect to the volume of TCAIM research being published each year over the past decades. With respect to the number of publications captured, although Danell et al.’s (2020) study was published in 2020, their coverage of the TCAIM literature only extended up until 2016 [34]. In the present study, over 37,000 publications were found to be published between 2017 and April 2021, comprising over 20% of the entire body of literature analysed.

### Future directions

Beyond the aforementioned comparative literature, it is worth noting that it has been far more common for bibliometric analyses to be conducted on a specific TCAIM-related topic. These have included acupuncture [54–56], aromatherapy [57], apitherapy [58], complementary and integrative oncology [59], ethnopharmacology [60], homeopathy [61], medicinal plants [62], qi gong [63], and yoga [64, 65], as just some examples among others. Others have conducted bibliometric analyses specific to methodologies, such as clinical trials [64, 66, 67] or guidelines [55] in TCAIM. Bibliometric analyses of the TCAIM literature with specific sub-topics are more straightforward to conduct, as the keywords and searches applied are likewise also easier to standardize. One of the main challenges in conducting comprehensive bibliometric analyses of the TCAIM literature in its entirety is the fact that it is very difficult to operationalize a dynamic and unrelated group of therapies that have been defined on the basis that they lie outside of the purview of conventional Western medical care [68, 69]. As a result, all of the bibliometric analyses of the TCAIM literature to date have been based on searches of TCAIM-specific journals or TCAIM-specific indexed headings, both of which unquestionably provide an incomplete picture of all the TCAIM literature. Thus, future directions of value include 1) the creation of an operational definition of TCAIM informed by a systematic search strategy, and 2) the development of standardized search strategies for major academic databases based on this operational definition.

### Strengths and limitations

This present bibliometric study captured and analysed the characteristics of over 170,000 publications, making it the largest conducted to date with respect to the TCAIM literature, and the most comprehensive with regards to TCAIM journal inclusion. Searches were conducted on Scopus as this academic database has a larger coverage in comparison to other databases such as Web of Science. Despite this, it must be acknowledged that all academic databases contain gaps in their indexing, and this was realized at the point of analysis in the present study. Publication data collected from Scopus was not externally verified against another source, and it is also important to note that the number of publications reflect what was indexed by the database as of the search date, and not necessarily the true number of publications published by the included journals themselves. It should be noted that publications included in this bibliometric analysis were based on the fact that they were published in a journal belonging to the “complementary and alternative medicine” category (code 2707), identified based on the ASJC provided by Scopus; as evidenced by Table 2, certain journals that changed names over their history were either not indexed in Scopus or were not included in the same ASJC category. Furthermore, it is always possible that some literature may not have been captured by not searching other databases, however, this would have introduced considerable complexities with respect to the ability to analyse search results efficiently (i.e. deduplication of such a large volume of publications, bibliometric network visualizations). The use of the software tool VOSviewer to create and visualize bibliometric networks serves as an additional strength to the present study, providing a deeper layer of analysis with respect to the strength and nature of relationships between different items (countries, keywords, authors, journals). Two final limitations include the fact that independent search results were extracted and analysed by a single author, and therefore, were prone to increased error as opposed to had the analysis been conducted in duplicate; additionally, results were not screened as this would have been impractical, and possibly unfeasible without the application of an operational definition of TCAIM. Without doing this, however, it is possible that this analysis also included non-TCAIM literature published in journals categorized as “complementary and alternative medicine” by Scopus.

## Conclusions

The present study provides current insight into the characteristics of publications published across TCAIM journals, and represents the largest bibliometric analysis conducted to date with respect to the TCAIM literature. The most productive countries included China, the United States, and Germany; unsurprisingly, a large proportion of common institutional affiliations and funding sponsors associated with this subset of publications also originated from these countries. The volume of publications has increased steadily since the 1940s, and a steep increase was observed between the mid-2000s and mid-2010s, which is largely attributable to increased available funding for TCAIM research globally. This upward trend has continued with 2020 marking the year with the most publications to date. Beyond identifying the large diversity of TCAIMs studied, this study also highlights therapies which may be understudied and warrant further investigation. Given a high prevalence of TCAIM use among patients, increased acceptance of TCAIM among conventional healthcare providers, and growing interest in the research of TCAIM, future work should continue to investigate and track changes in the publication characteristics of the emerging research on this topic. The creation of an operational definition of TCAIM informed by a systematic search strategy, followed by the development of standardized search strategies for major academic databases based on this operational definition, may serve to achieve these goals more comprehensively.

## Data Availability

All data generated or analysed during this study are included in this published article.

## Abbreviations

ASJC: All Science Journal Classification
TCAIM: Complementary, alternative, and integrative medicine
NCCIH: National Center for Complementary and Integrative Health
